# Supplementary material for: Transluminal Pillars—Their Origin and Role in the Remodelling of the Zebrafish Caudal Vein Plexus
Source: Int J Mol Sci. 2023 Nov 24;24(23):16703. doi: 10.3390/ijms242316703 (PMC10706262; doi:10.3390/ijms242316703)
Supplement: Supplementary file 1 [file ijms-24-16703-s001.zip › Suppl Figures.pdf]

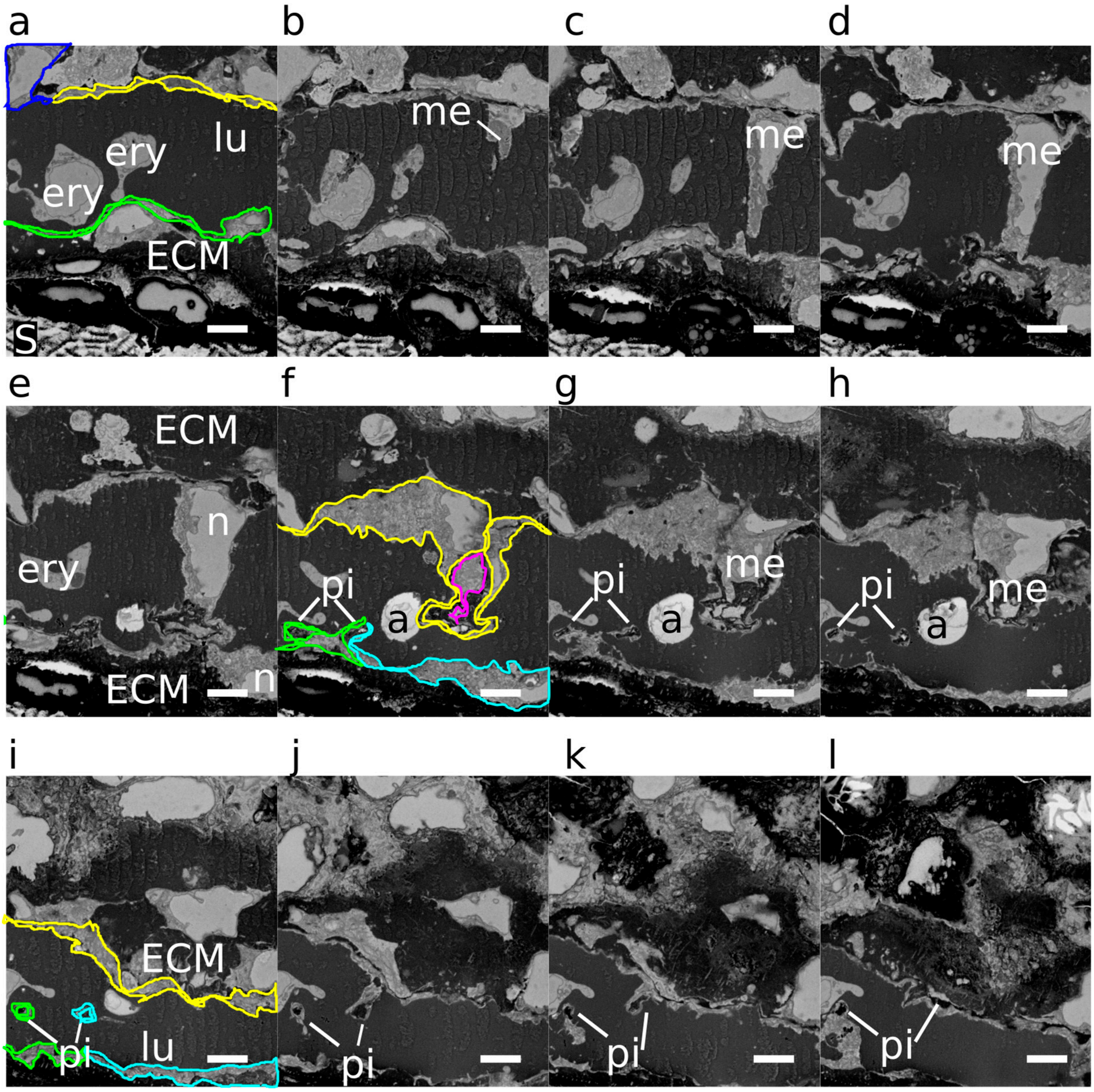

Suppl. Fig. S1: Ultrastructure and 3D reconstruction of a pillar and a mesh (3View data)
Serial block face EM sections showing the difference between a mesh and a pillar. Lateral view of a thin vessel in the ventral part of CVP. The lumen (lu) surrounded by a thin endothelial layer has a uniform gray color, with nucleated erythrocytes (ery) being present on the first sections. The outline of single endothelial cells are shown in yellow, green, purple, turquoise (a, f, i): the outline in magenta marks a perivascular cell whose processes may be seen inside of the mesh, surrounded by an EC in yellow (f). The surrounding loose ECM is dark and contains perivascular cells.

A mesh (me) containing an endothelial cell and some ECM components can be visible on sections b-j. The mesh is ca 6μm in diameter, consists of an endothelial cell (EC) and in its core, a nucleus is clearly visible. Besides the cellular component, some ECM is present. In contrast to that, two pillars (pi, sections f-l) contain merely a core formed by electron dense ECM, sometimes with collagen (whitish substance), surrounded by a thin cellular coat from an endothelial cell. The structure marked with "a" could not be identified and is likely and artifact. Sections a, f and i: different endothelial cells are shown in yellow, green, cyan and blue and a perivascular cell invading the mesh is shown in magenta. Selected slices are ca 0.6μm apart (range 0.46-0.84μm), scale bar: 5μm.

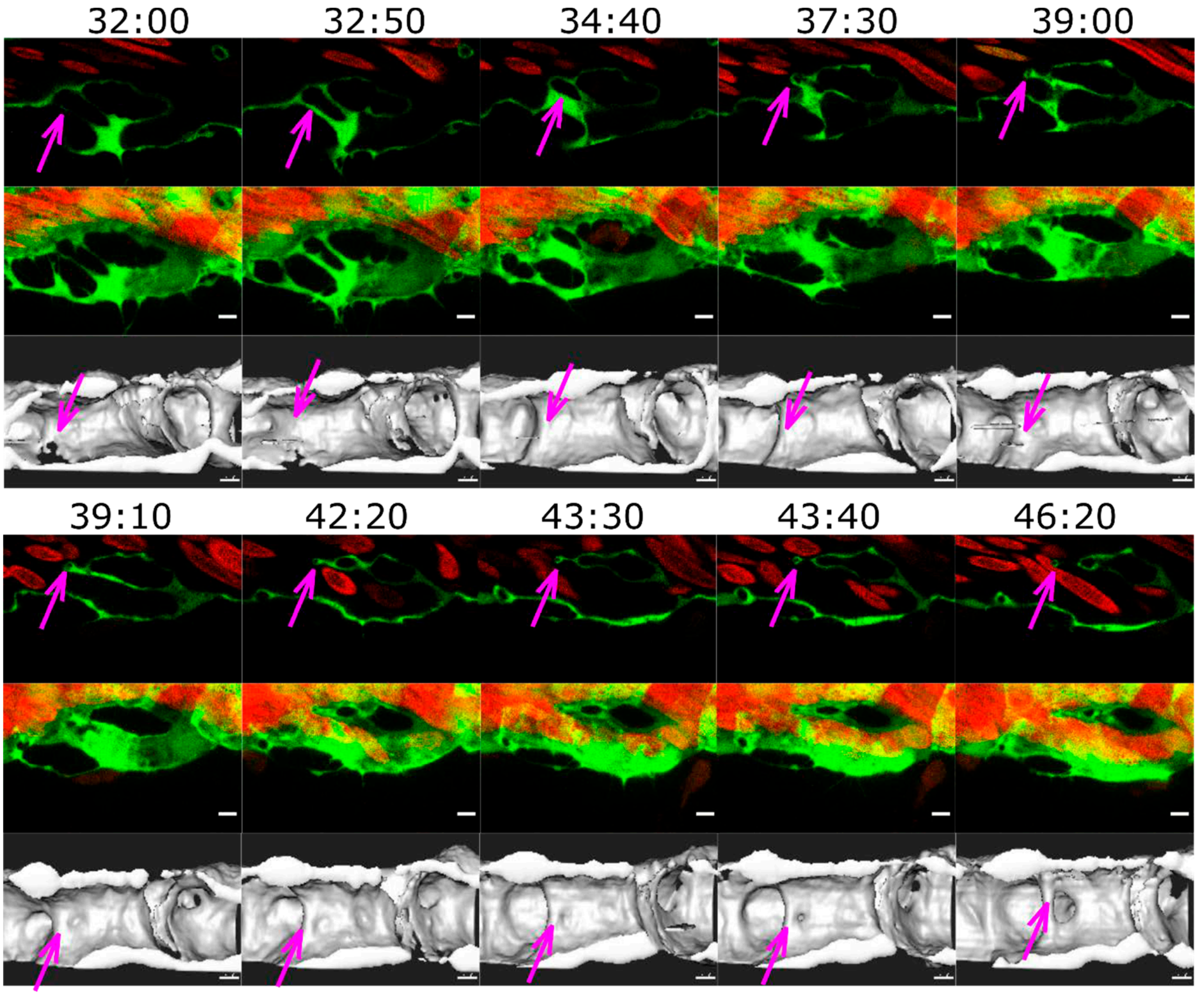

Suppl. Fig. S2: Formation of a pillar by sprouting angiogenesis.
At 32:00, numerous filopodia are present and via their contact, meshes form (32:50-37:30). One of them gives rise to an oval-shape structure that eventually divides the vessel into two branches (39:00-42:00), the second one disappears again, the retraction of the filopodia is visible (37:30-42:20). A pillar (position indicated by a magenta arrow) splits off the oval mesh (43:30-46:20). The origins of the pillar core can be tracked back to the sprouting phase – the future core is delineated by the filopodia.
Endothelium in green (eGFP), erythrocytes in red (dsRed), bright field in grey. First row: single slice (5µm thick); second row: maximum intensity projection (only green and red channel); third row: surface model in grey (based on the GFP channel). Time points (hh:mm post fertilization) valid for each column, scale bar: 5µm.

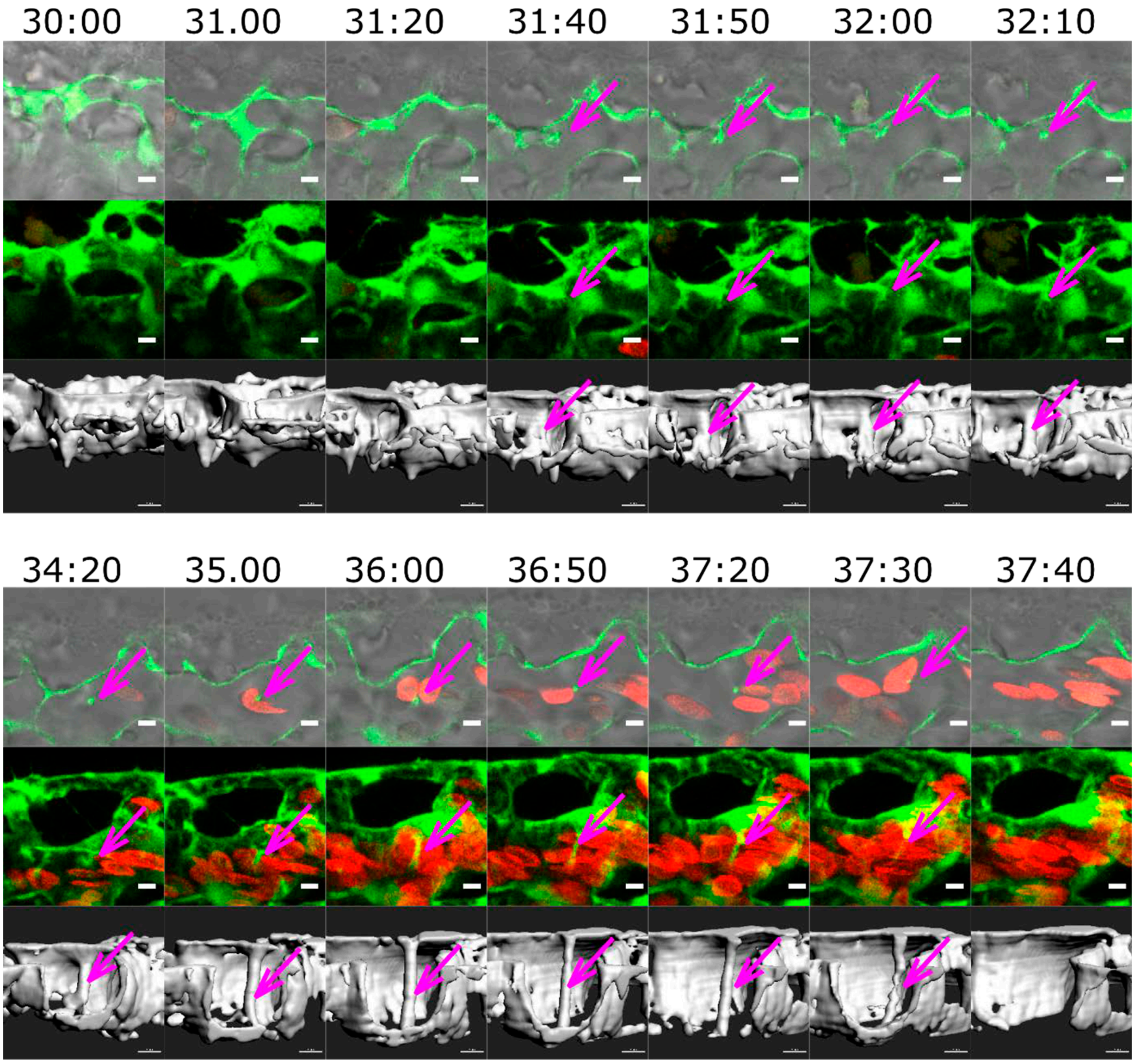

Suppl. Fig. S3: Formation of a pillar and subsequent rupture.

Mechanism of pillar formation (magenta arrows): lumen expansion/sprouting angiogenesis. Initially, the vessel is only partially lumenised, the pillar emerges as the lumen fully inflates and the vessel is perfused. The pillar first appears in the lumen as a fold (31:40-32:00). The pillar core is thin but present (31:40, 36:00), seems to disappear later (37:30) which weakens the pillar, and it ruptures within next 10 minutes (until 37:40).

Endothelium in green (eGFP), erythrocytes in red (dsRed), bright field in grey. First row: single slice (5µm thick); second row: maximum intensity projection (only green and red channel); third row: surface model in grey (based on the GFP channel). Time points (hh:mm post fertilization) valid for each column, scale bar: 5µm.

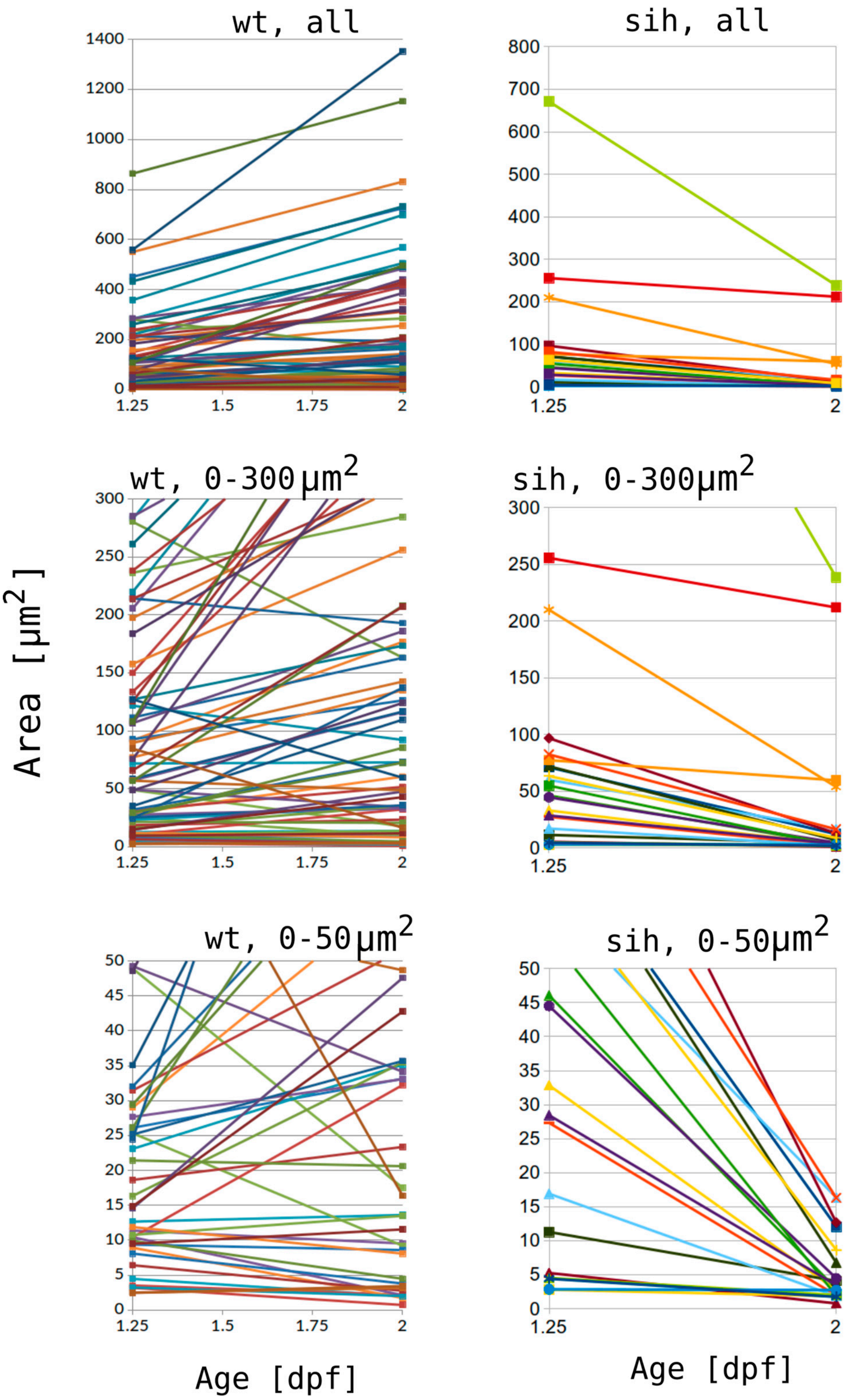

Suppl. Fig. S4 Absolute cross-section area change of pillars and meshes in wt and *sih* embryos.
The initial measurements took place at 1.25 dpf, the second measurements at 2 dpf, each line connects a pair of measurements in these two time points, the symbols and colors were used for better visibility of the individual measurement and have no special meening. Top row: Overall, there was a tendency of growth of the structures in wt and shrinkage in *sih* embryos. Middle row: medium-sized structures (0-300 µm^2) also tended to grow further in wt, whereas the opposite was true for the meshes observed in *sih* embryos.

Bottom row: small structures (0-50 µm^2) were very variable in their behaviour in wt, with both growing and shrinking structures. In *sih*, all the measured structures shrank within the observed time span.

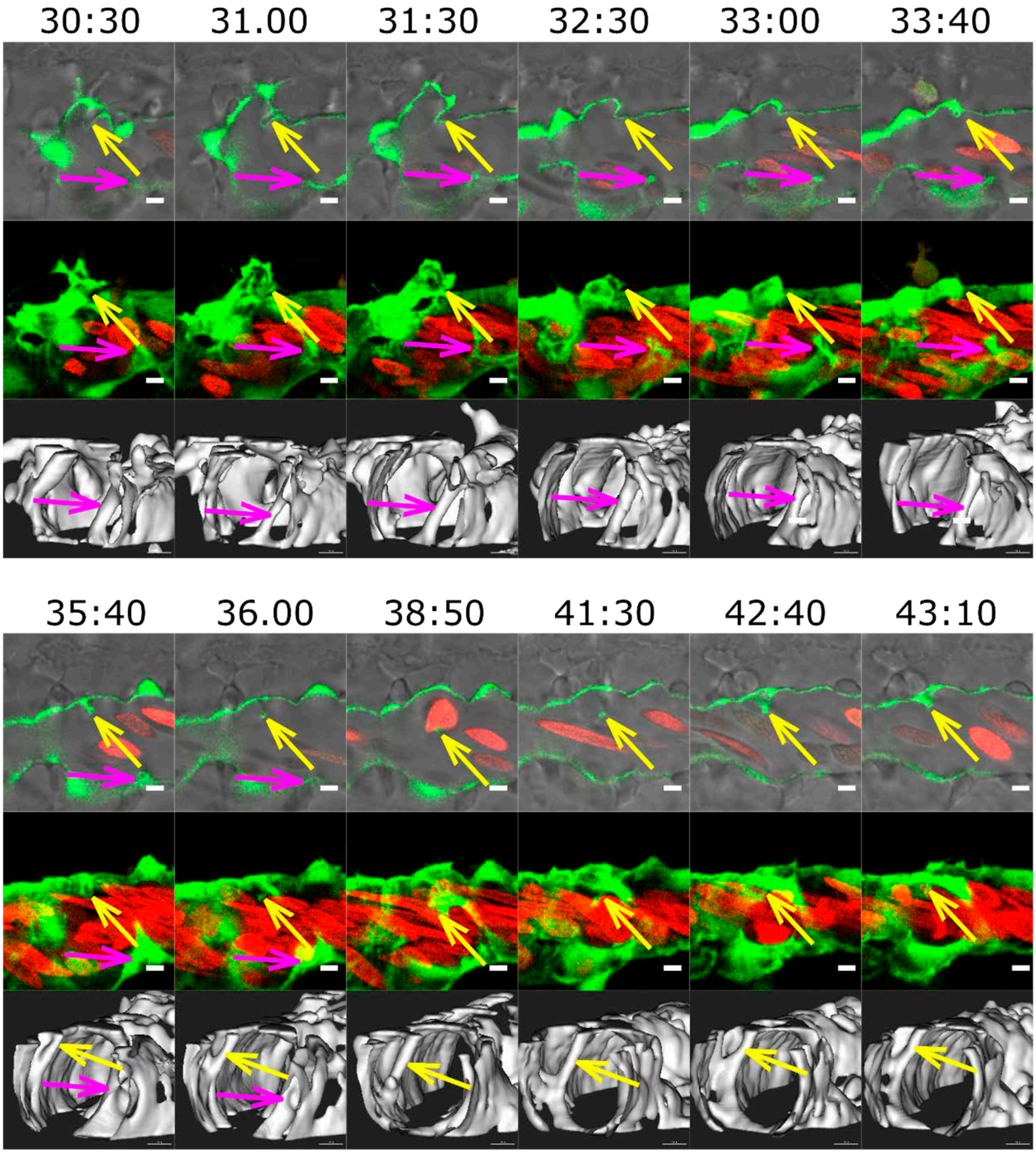

Suppl. Fig. S5: Formation of a pillar and its disappearance by merging with the vessel wall.
Mechanism of pillar formation (yellow arrows): capillary wall folding. At the beginning, the vessel is already lumenised and there is active sprouting. The pillar forms later when the outer vessel surface looks smooth, not sprouting anymore. First, a fold appears (32:30). As the blood cells enter the vessel and its diameter expands, the fold closes around the pillar core (33:40-35:40), and eventually the pillar enters the lumen (36:00). Later the pillar moves back to the vessel wall where it originated from and fuses with it again (43:10). Another pillar (magenta arrows), that has formed before 30:30, also disappears via this mechanism (36:00).
Endothelium in green (eGFP), erythrocytes in red (dsRed), bright field in grey. First row: single slice (5µm thick); second row: maximum intensity projection (only green and red channel); third row: surface model in grey (based on the GFP channel). Time points (hh:mm post fertilization) valid for each column, scale bar: 5µm.

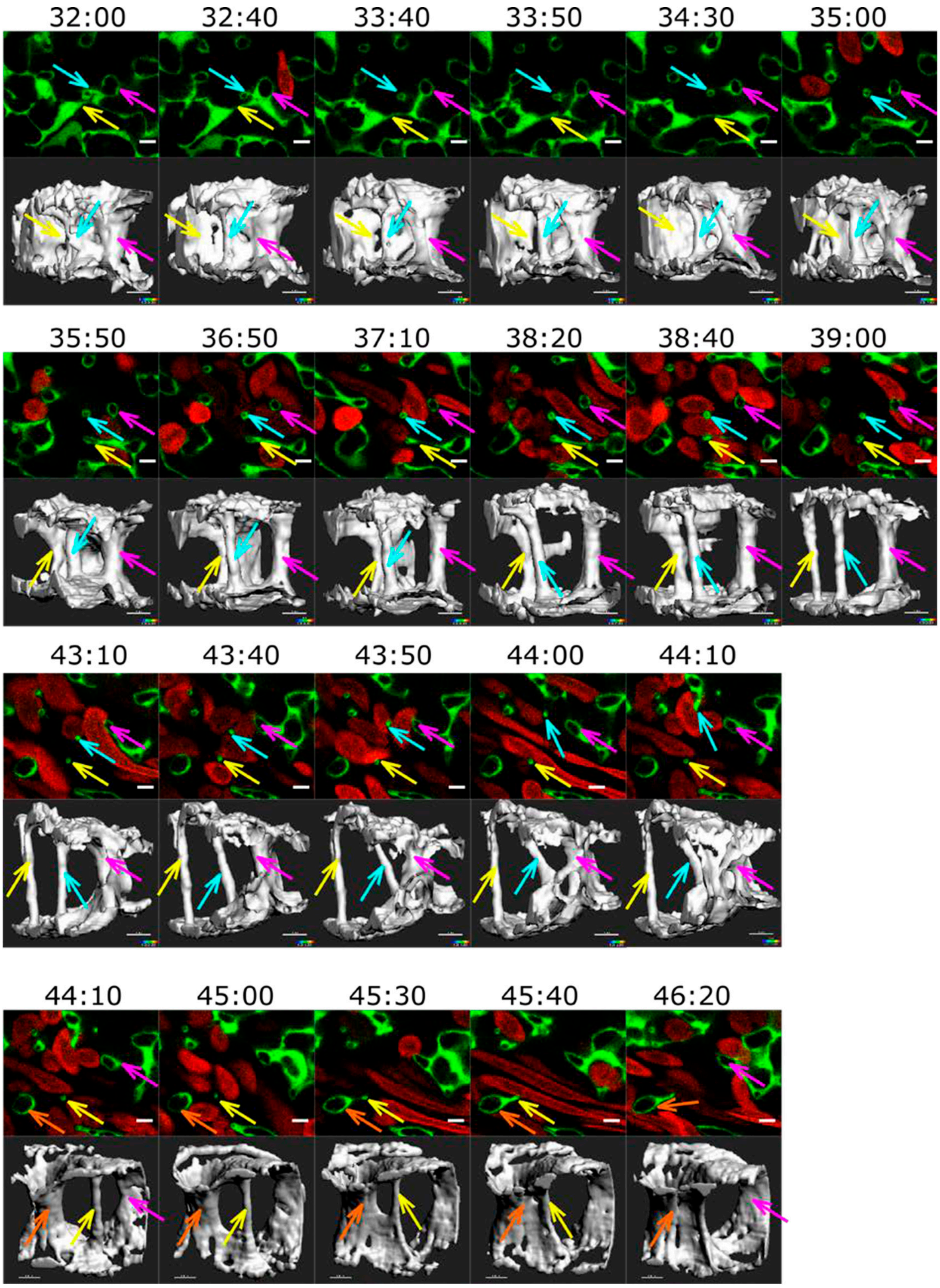

Suppl. Fig. S6: Pillar behaviour.

Mechanism of formation: possibly by lumen expansion/sprouting (yellow, magenta and cyan arrows, not tracked from the very beginning). At 32:00, the future cores of two pillars (magenta and cyan) are clearly visible. At this time point the vessel is already partially lumenised and there is active sprouting that possibly formed the basis of those future pillars. However, the major force in pillar formation is lumen expansion. The pillars appear as the connection between their folds is perforated (32:40 -35:00). The pillar marked with the yellow arrow splits from a long intraluminal fold (32:00-34:30) that evolved into a longitudinal mesh (35:00-37:10), with a transient presence of an endothelial bridge connecting the yellow

pillar with the other half of the mesh (38:20-38:40). The pillar marked with magenta partially merges with a neighbouring mesh (43:10 until 46:20) but they do not form a single large mesh. The pillar marked with cyan gets thinner (it seems as if its core has disappeared), moves away (43:10-44:00) and eventually merges with a nearby mesh (44:10). The pillar marked with yellow follows the same fate (41:00- 46:20, mesh marked with an orange arrow).
Endothelium in green (eGFP), erythrocytes in red (dsRed). First row: single slice (5µm thick); second row: surface model in grey (based on the GFP channel). Time points (hh:mm post fertilization) valid for each column, scale bar: 5µm.

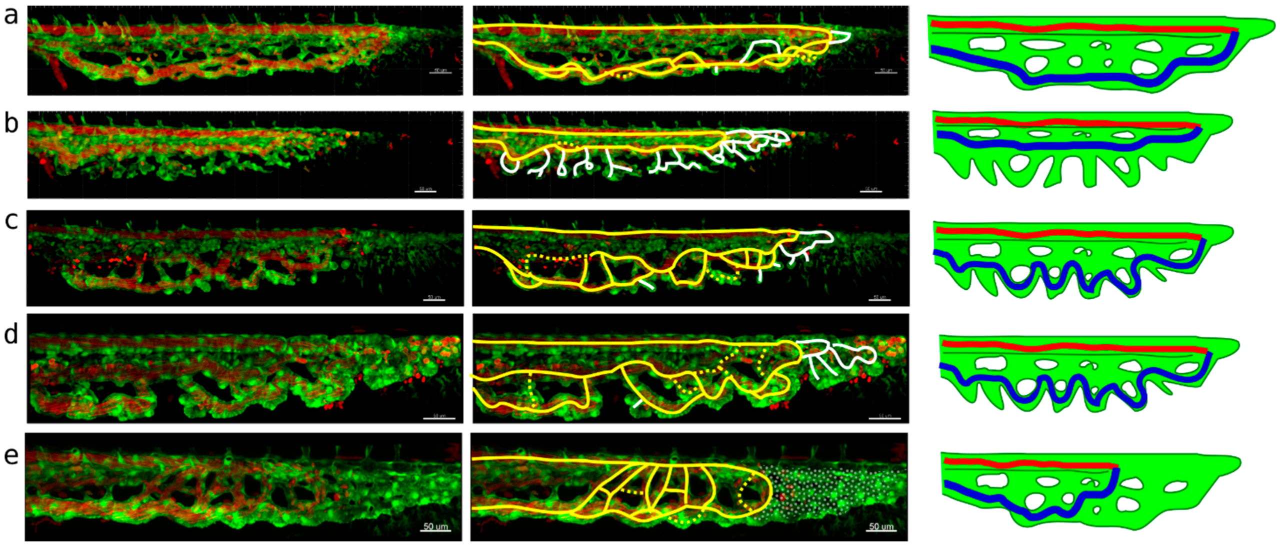

Suppl. Fig. S7: Preliminary data on the effect of different inhibitors on CVP morphology.
Left side: maximal intensity projection of confocal images taken at 48 hpf. Green: endothelium (eGFP), red: erythrocytes (DSred). Middle: the vessel loop is shown in yellow, dashed lines show only sparsely perfused regions, continuous lines the major route(s) of blood flow, pillars and blind-ending vessels are depicted in white (dots and lines, respectively). Right side: schematic drawing of the different CVP-patterns, DA in red, CV in blue, endothelium in green. In general, the plexus appeared underdeveloped at 48 hpf. The DA was not completely separated from the venous part, resulting in a distal non-perfused region. The pillar formation itself was as well dominated by the lumen expansion/sprouting mechanism and was not different from what we observed in control embryos. (a) Control embryo (wt). Establishment of a loop between DA and CV can be well seen, in the area of the CV there are a few vessel loops left. (b) CVP of an embryo treated with Latrunculin B, an inhibitor of filopodia formation since 24 hpf. The CVP is smaller in its antero-posterior as well as its cranio-caudal dimensions, the meshes are bigger and there are numerous lumenised, but blind-ending vessels in the venous part (white lines). (c) CVP of an embryo treated with sorafenib, a VEGFR2 inhibitor, since 24 hpf. The dimensions of the CVP are similar to the wt control, but there are fewer, larger meshes. There are persistent venous vessel loops and some blind-ending venous segments, so a clear course of the CV cannot be identified. (d) CVP of an embryo treated with cediranib, also a VEGFR2 inhibitor, since 24 hpf. The plexus appearance is comparable to the treatment with sorafenib with tortuous veins visible in the venous part of the CVP. (e) CVP of an embryo treated with BHG712, an EphB4 inhibitor, since 24 hpf. This is the most notably changed phenotype. The plexus is shorter in its cranio-caudal dimensions, only around 2/3 of the plexus are perfused. There are several connections between the DA and the CV and in the most caudal part, which seemed empty, numerous pillars can be seen. In those embryos, the most cranial connections between DA and CV remained open while the plexus still grew in caudal direction. Therefore, the loop formed by the blood flow changed only minimally in treated embryos, while the blood flow gradually expanded more caudally in controls.

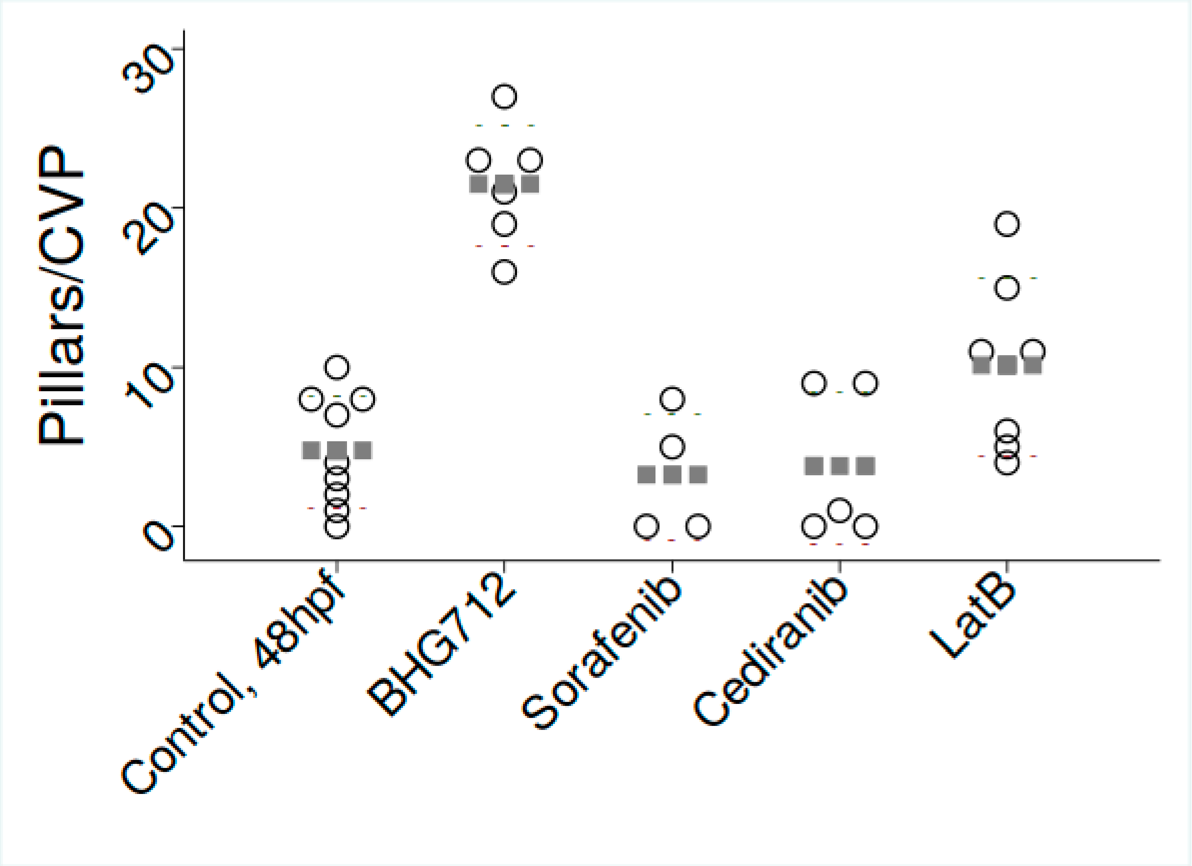

Suppl. Fig. S8. Effect of inhibitor treatment on pillar count. The number of pillars per plexus counted at 48 hpf can be seen for embryos treated with BHG712, sorafenib, cediranib and LatB, respectively. Each measurement is represented with a circle, the grey squares indicate mean, dashes mean plus or minus standard deviation. Treatment started at 24 hpf for all the inhibitors. An increased number of pillars could be seen in the treatment with BHG712 as well as LatB. Treatment with both sorafenib and cediranib resulted in a pillar number that was similar to wt controls.

Suppl. Video. S1
Formation of pillars in the ventral part of CVP of a Fli:eGFP/Gata:dsRed embryo, green=endothelium, red=erythrocytes. Time 00:00 starts at 32hpf, the region corresponds the surroundings of the ROI that we show in more detail in Fig.1. The plexus expands via SA, the sprouts are at first not lumenized but gradually, the lumen expands and eventually is perfused. Please observe the pillar appearande as the lumen forms, and the rather passive behavior of already existing pillars. Scale bar 10μm.

Suppl. Video. S2
Pillar formation of sprouting angiogenesis in more detail, different embryo than in Video 1 but the same model Fli:eGFP/Gata:dsRed. Time 00:00 corresponds to 30:50hpf, the region corresponds to the ROI in Fig. 3. Observe the interaction of sprouts and the emergence of the two pillars and their behavior. Scale bar 10μm.

Suppl. Video. S3
Formation of a pillar in dorsal aorta, again the Fli:eGFP/Gata:dsRed model, gray=brightfield channel. Time 00:00 corresponds to 32hpf, the region shows the caudal part of CVP, above in the middle is the ROI shown in more detail in Fig. 4. Observe the intraluminal fold in DA, from which gradually a transversal pillar evolves.

Suppl. Video. S4
CVP development in a sih/Fli:eGFP embryo. Time 00:00 corresponds to 30hpf, the region corresponds to a central part of CVP, in the middle being the ROI shown in more detail in Fig. 5. Please observe the formation of tiny pillars and shrinking of the present meshes. Scale bar: 10μm.

Suppl. Video. S5
Almost the whole CVP of a Fli:eGFP/Gata:dsRed embryo (identical as in V1), green=endothelium, red=erythrocytes, time 00:00 starts at 32hpf. Observe the splitting of the meshes in the caudal part and arteriovenous splitting which is in more detail shown in Fig. 7.
